# Philadelphia Academy of Surgery

**Published:** 1894-08

**Authors:** William Hunt

**Affiliations:** The President, in the chair


### Society Reports.

### PHILADELPHIA ACADEMY OF SURGERY.

### MEETING JUNE 4, 1894.

*The President, Dr. William Hunt, in the chair.*

RICHARD H. HARTE, M.D., Surgeon to the Pennsylvania and Episcopal Hospitals, reported "A Case of Pyemia Due to Appendicitis."

The history of the case I wish to present you this evening is as follows: A. C., aged twenty-five years, a weaver by occupation, was admitted to the medical wards of the Episcopal Hospital, May 9, 1894, at the request of his medical attendant, Dr. Ferguson, supposing the man to be suffering from abscess of the liver.

On admission the following facts were elicited, which I have copied from the Resident's meager notes; *Family history,* negative. *Previous history:* Enjoyed good health, although not especially robust; about three years ago recalls having a short illness ushered in by a chill, the prominent symptoms of which were sharp cramp-like pains referred to the lower third of the abdomen; was confined to bed for a week. (This was undoubtedly an attack of appendictis.) *Present attack:* States that he was feeling perfectly well up to about two weeks ago, when he was awakened with sharp pains in the right iliac fossa, and in the course of the morning they were followed by a pronounced chill, succeeded by a sweating; through the day he felt nauseated, and in the evening vomited.

During the interval of two weeks from the time of his first attack until his first admission into the hospital, he had always once in the twenty-four hours, and sometimes oftener, a decided chill followed by profuse sweating; pain, referred in the right iliac, umbilical and hypochondriac regions, was almost continuous; the bowels were watery, and moved daily; the patient was confined to bed and growing weaker.

After his admission into the medical wards all his symptoms were referred to the region of the liver, over which there was distinct tenderness. The daily chill and high temperature (106° F.) naturally led my colleague, Dr. Morris, on the medical side, to suspect abscess of the liver, and he transferred the case to the surgical wards for operation.

On the day after his transfer to the surgical wards I found the case very much as above stated, the right hypochondriac region being tender on pressure, liver area increased. On examining the case in the ward before operation, I exposed but a small portion of the abdomen and noticed a distinct eruption, which I supposed was due to the vigorous use of a scrubbing brush. The case was then taken to the operating-room and etherized, and on a more careful examination of the abdomen under an anesthetic, I found the eruption, which I had first supposed was due to the bichloride and friction, to be pretty generally distributed over the entire trunk, and in appearance was not unlike the eruption of typhus fever, or, in other words, a distinctly morbilliform eruption. The history at that time in my possession was rather negative, and I decided not to operate until more definite data could be obtained. On the following day I saw the physician under whose care he had been, and with my colleagues, Drs. Deaver, Neilson and Morris, decided to make an exploratory incision over the region of the liver. The patient was etherized, and an incision corresponding to the right semi-lunar line gave a free opportunity to explore the surface of the liver, which appeared normal. An exploration with an aspirating needle failed to reveal any purulent collections. The region of the appendix was explored through the abdominal wound, suspecting that possibly it might be the seat of the trouble; but with the hand carried down over the liver to the right iliac fossa, no evidence of trouble was apparent.

After the operation the chills seemed to be less severe, not being so frequent as before, and the temperature not rising so high. The external wound closed quickly, and no symptoms relative to the operation were manifest. The next chill was four days after the operation, and did not rise nearly to within two degrees of the height of the previous one. The next chill did not appear until the fifth day, although the patient was gradually growing weaker, and died on the tenth day after the operation. After the second chill he began to expectorate bloody mucus, sometimes a cupful of blood being expectorated during the twenty-four hours.

A *post mortem* examination revealed the liver slightly enlarged and filled with a large number of metastatic abscesses, the principal pus collection and largest abscess being in the left lobe. The appendix was entirely destroyed, and its position occupied by a small pus cavity holding about three drachms of pus. The cecum for several inches beyond its attachment to the appendix was gangrenous. There were some septic deposits in the lungs, although no distinct infarcts were to be found. The reason I ascribe for the liver being affected, which is usually a secondary affection coming under the general circulation, is that the *materies morbi* coming from the appendix immediataly entered the portal circulation—superior mesenteric vein—and consequently the first deposit would naturally be found in the liver.

The *post mortem* here distinctly revealed a case of pyemia, the primary cause of infection arising from the appendix. One peculiar feature in the case was the eruptions, which were more or less misleading, although eruptions in suppurative fever, or pyemia, have long been recognized, and are spoken of by Braidwood in his exhaustive treatise on that disease.

My object in briefly reporting this case to-night, is that I think it of no little interest (without wishing to go into the subject of pyemia which is so familiar to all the Fellows of the Society), throwing as it does more light upon the much mooted subject of appendicitis, and again, adding its weight to the testimony that the above mentioned disease is strictly a surgical affection rather than a medical one; for I feel certain that had the true condition of affairs been recognized at the onset of the attack the ultimate termination might have been different.

DISCUSSION.

DR. WILLIAM J. TAYLOR: Some ten years ago I made a *post mortem* examination for Dr. William Moss, of Chestnut Hill.

At that time we did not know very much about inflammation of the appendix. There was a long-standing condition which was supposed to be an ulcerated condition of the bowel. It was undoubtedly due to inflammation around the appendix. There were also pyemic abscesses throughout the liver. The patient, a boy fourteen years of age, had died of general pyemia due to infection from this inflammatory condition of the appendix.

DR. T. S. K. MORTON: I have never met with pyemia where abscess about the appendix was found, but I recall a number of instances in literature where pylephlebitis had extended from the abscess cavity directly to the liver. I have seen one or two cases where intense jaundice accompanied this condition. One of these cases was in the practice of Dr. Steinbach. Here it was quite evident that the infection about the appendix had traveled by the lymphatic or venous route to the liver.

THE PRESIDENT: Pyemia associated with appendicitis must be a rare condition. I do not recall having seen it. I take it that those cases in which there is distinct evidence of involvement of the liver are essentially fatal.

DR. L. W. STIENBACH: The case referred to was one of appendicitis in a young man. I saw him for the first time on the fifth or sixth day of the disease. Slight jaundice was present at the time of operation, but increased rapidly after the operation. The patient lived a week or ten days after the operation.

C. B. PENROSE, M.D., read a paper entitled "Use of the Abdominial Drainage-Tube Determined by Bacteriological Examination."

The subject of drainage in abdominal surgery is one about which there is still great difference of opinion, though this difference is much less than it was only a few years ago. Some operators never used drainage at all after any operations, and yet obtained exceedingly good results; while others obtained equal results, and their statistics showed that they employed drainage in a proportion of their cases, which varied, according to the individual taste of the operator, from five or ten per cent. to seventy-five per cent. The general advice given by the advocates of drainage was "When in doubt drain." It was this element of doubt which caused the diversity of practice. The doubting operators drained the most. Everything which increases our knowledge in regard to the facts which determine drainage diminishes our doubts and brings about more uniformity of practice.

We drain the abdomen for two reasons—for hemorrhage and for septic material. As the experience of the operator increases and his skill in enucleating tumors becomes greater, he has less hemorrhage, and, other things being equal, he drains less. Our methods of controlling hemorrhage in abdominal operations are better than they were a few years ago, the Trendelenburg posture enabling us to check bleeding from the small vessels in the bottom of the pelvis, which before the introduction of this position required drainage. The operator who enucleates pelvic tumors with two fingers, and closes the abdomen without seeing what he has done, will necessarily have much more doubt in regard to hemorrhage, and will use the drainage-tube much more frequently than the operator who inspects the field of enucleation before closing the abdomen.

The second reason for drainage is the septic character of the material which escapes or is retained in the abdomen. Knowledge in regard to this fact is of great value in deciding about drainage in any case.

During the past winter, at the University Hospital, an immediate bacteriological examination has been made of the contents of every tubal or ovarian tumor which was ruptured during removal. And the report of the pathologist in regard to the septic or the aseptic character of the contents has determined my decision in regard to the use of the drainage-tube.

I, unfortunately, have no record of the total number of cases in which such examinations have been made; but the results have been exceedingly satisfactory, for out of a series of forty-six celiotomies, in which drainage was used but three or four times for hemorrhage, and only once because the microscope showed the material which escaped into the abdomen to be septic, there was no case of peritonitis or sepsis.

The tubal contents in most cases of salpingitis are sterile. Shauta (*Archiv für Gynecologie*, 1893, No. 44) reports 192 cases of salpingitis, in 144 of which the contents of the tubes were sterile, in 33 there were gonococci, and in 15 streptococci or staphylococci.

Before I began to use this method of bacteriological examination I inserted a drainage-tube in every case of tubal and ovarian abscess where the contents escaped into the peritoneum. Now I neither irrigate nor use the drainage-tube unless the microscope shows these contents to be septic. The presence of gonococci in small numbers does not necessitate drainage. Recently the value of this bacteriological examination was illustrated by two cases operated on consecutively. Each woman had a tubo-ovarian abscess, caused by sepsis at labor. In each case the abscess was ruptured during removal, and the pelvis filled with pus. In the first the pus was found to be sterile, and I closed the abdomen without irrigation or drainage. In the second one pus contained streptococci and staphylococci and cola commune. Consequently, the pelvis was thoroughly washed out and drained.

Both women recovered without peritonitis or sepsis, though the convalescence of the first was very much easier than that of the second.

The examinations have been made for me by Dr. Beyea. Cover-glass preparations of the material to be examined are made and are fixed in the flame of an alcohol lamp, and stained with carbol-fuchsin. The microscopic examination is made with a Leitz 1.12 immersion lens.

The examination is quickly and easily made, and I think that no operating-room is completely equipped without facilities for such bacteriological examinations. They furnish us with scientific data from which we can determine the propriety of an important surgical procedure, which otherwise depends upon the whim or prejudice of the operator.

### DISCUSSION.

DR. J. EWING MEARS: I think Dr. Penrose has made a very valuable contribution to the literature of this subject. I am reminded of some work of a similar character which has been done by Dr. L. McLane Tiffany, of Baltimore. At the recent meeting of the American Surgical Association, in the discussion on empyema, he stated it was his custom to submit the fluids removed from the chest cavity to bacteriological examination, and the question of the kind of operation and washing out of the cavity was determined by the result of this examination. The value of this work cannot be overestimated. It enables us to make our diagnosis positive, and determines the character of treatment to be employed.

DR. RICHARD H. HARTE: I believe with Dr. Penrose that no operating-room is complete without a suitable bacteriological plant. At the Pennsylvania Hospital, with the assistance of the pathologist and one of the residents, I have been trying to do some work of this character, and have derived a great deal of assistance from it. I notice in a number of operating-rooms in new hospitals that a bacteriological-room is also provided. A great deal of information as to the necessity for drainage can be obtained in this way.

---

### LOVE AND LAUGHTER.

Laugh and the world laughs with you,
Weep, and you weep alone;
This grand old earth must borrow its mirth,
It has sorrow enough of its own.

Sing, and the hills will answer,
Sigh, it is lost on the air;
The echoes bound to a joyful sound,
But shrink from voicing care.

Be glad, and your friends are many,
Be sad, and you loose them all;
There are none to decline your nectared wine,
But alone you must drink life's gall.

There's room in the halls of pleasure
For a long and lordly train,
But one by one we must all file on
Through the narrow aisle of pain.

Feast, and your halls are crowded,
Fast, and the world goes by;
Succeed and give, 'twill help you live,
But no one can help you die.

Rejoice, and men will seek you,
Grieve, and they turn and go;
They want full measure of all your pleasure,
But they do not want your woe.

—*John A. Joyce.*

